# Dimerization Mediates Thermo-Adaptation, Substrate Affinity and Transglycosylation in a Highly Thermostable Maltogenic Amylase of *Geobacillus thermoleovorans*

**DOI:** 10.1371/journal.pone.0073612

**Published:** 2013-09-19

**Authors:** Deepika Mehta, Tulasi Satyanarayana

**Affiliations:** Department of Microbiology, University of Delhi South Campus, New Delhi, India; University of South Florida College of Medicine, United States of America

## Abstract

**Background:**

Maltogenic amylases belong to a subclass of cyclodextrin-hydrolyzing enzymes and hydrolyze cyclodextrins more efficiently than starch unlike typical α-amylases. Several bacterial malto-genic amylases with temperature optima of 40–60°C have been previously characterized. The thermo-adaption, substrate preferences and transglycosylation aspects of extremely thermostable bacterial maltogenic amylases have not yet been reported.

**Methodology/Principal Findings:**

The recombinant monomeric and dimeric forms of maltogenic α-amylase (Gt-Mamy) of the extremely thermophilic bacterium *Geobacillus thermoleovorans* are of 72.5 and 145 kDa, which are active optimally at 80°C. Extreme thermostability of this enzyme has been explained by analyzing far-UV CD spectra. Dimerization increases T_1/2_ of Gt-Mamy from 8.2 h to 12.63 h at 90°C and mediates its enthalpy-driven conformational thermostabilization. Furthermore, dime-rization regulates preferential substrate binding of the enzyme. The substrate preference switching of Gt-Mamy upon dimerization has been confirmed from the substrate-binding affinities of the enzyme for various high and low molecular weight substrates. There is an alteration in K_m_ and substrate hydrolysis efficiency (V_max_/K_m_) of the enzyme (for cyclodex-trins/starch) upon dimerization. N-terminal truncation indicated the role of N-terminal 128 amino acids in the thermostabilization and modulation of substrate-binding affinity. This has been confirmed by molecular docking of β-cyclodextrin to Gt-Mamy that indicated the requirement of homodimer formation by the interaction of a few N-terminal residues of chain A with the catalytic residues of (α/β)_8_ barrel of chain B and vice-versa for stable cyclodextrin binding. Site directed mutagenesis provided evidence for the role of N-terminal D109 at the dimeric interface in substrate affinity modulation and thermostabilization. The dimeric Gt-Mamy transglycosylates hydrolytic products of G4/G5 and acarbose, while the truncated form does not because of the lack of extra sugar-binding space formed due to dimerization.

**Conclusion/Significance:**

N-terminal domain controls enthalpy-driven thermostabilization, substrate-binding affinity and transglycosylation activity of Gt-Mamy by homodimer formation.

## Introduction

Cyclodextrins are the cyclic oligosaccharides of glucose linked via α-(1,4)-glycosidic linkages. Among carbohydrases that hydrolyze cyclodextrins are the maltogenic amylases (glucan-1,4-α-maltohydrolase, EC 3.2.1.133), which belong to a subclass of cyclodextrin hydrolyzing enzymes along with neopullulanases and cyclomaltodextrinases in the family13 (α-amylase family) of glycosyl hydrolases. A few properties make them distinguishable from the typical α-amylases. Firstly, they hydrolyze cyclodextrins more efficiently than starch and produce maltose and glucose as major hydrolytic products. Secondly, they are catalytically versatile, that is, they hydrolyze α-(1,4)- as well as α-(1,6)- linkages of the substrate molecule and transglycosylate the hydrolytic products. Thirdly, they possess an additional 130 residues at the N-terminus that are absent in the typical α-amylases. Fourthly, they can hydrolyze acarbose, a potent inhibitor of α-amylases, to produce glucose and acarviosine-glucose [pseudotrisacc-haride]. Maltogenic amylases are, therefore, known as multifunctional amylases [1].

To date maltogenic amylases with temperature optima of 40–60°C have been reported from the bacteria [2]–[6]. Only a few extremely thermostable maltogenic amylases have been reported from archaea with temperature optima in the range of 70–100°C [7]–[10]. This investigation is the first thorough insight into an extremely thermostable maltogenic amylase (T_opt_ 80°C) from the extremely thermophilic bacterium *Geobacillus thermoleovorans*. Higher thermostability of the dimeric maltogenic amylase of *G. thermoleovorans* (Gt-Mamy) than the known bacterial maltogenic amylases [2]–[6] encouraged us to investigate the role of dimerization as a thermo-adaptive mechanism. We have biochemically and biophysically characterized this enzyme and correlated dimeric state of Gt-Mamy to the substrate preference switch and transglycosylation activity by N-terminal truncation and site directed mutagenesis. To our knowledge, this is the first detailed investigation on the role of dimerization as a thermo-adaptive mechanism, regulation of substrate-preference switching and transglycosylation activity of a highly thermostable maltogenic amylase.

## Results and Discussion

### PCR amplification and sequence analysis of Gt-Mamy

Using the primers Mal_int_F/R, a ∼300 bp amplicon was sequenced, which shares 88–93% identity with the α-cyclodextrinase of *G. stearothermo-philus* (GenBank ID AB070710.1), α-amylase catalytic region of *Geobacillus* sp. Y412MC52 (GenBank ID CP002442.1), maltogenic amylase of *Geobacillus* sp. Gh6 (GenBank ID GQ884176.1) and maltogenic amylase of *G. thermoleovorans* (GenBank ID CP003125.1). Using the end regions of maltogenic amylase sequences and end regions of the region I (300 bp) sequence, external and internal primers were designed and PCR amplified and sequenced. All the sequences were overlapped for obtaining the full-length *Gt-Mamy* gene (Figure S1). The sequence analysis revealed an ORF of 1767 bp (GenBank ID JQ999960). The *in silico* translated protein sequence (588 amino acids) has the predicted pI and molecular mass of 5.52 and 68.5 kDa, respectively.

Sequence alignment with the closely related enzymes of GH-13 family (Figure S2) identified seven conserved regions (region I, II, III, IV, V, VI and VII) atypical of the members of α-amylase family (GH-13). These regions ^242^DAVFN^246^, ^324^GWRLDVAN^331^, ^351^VYILGEI-WH^359^, ^419^LLGSHD^424^, ^295^MPKLNT^300^, ^189^GITGIYLTP^197^, ^451^GSPCIYYGD^459^, lie in β3, β4, β5, β7, loop 3, β2 and β8 regions of the (β/α)_8_-barrel domain, respectively [11]. The comparison of Gt-Mamy with the Gt-amy (α-amylase of *G. thermoleovorans*) [12] showed the presence of extra N-terminal 128 amino acids (N-domain) in the former, a distinguishing feature of cyclodextrin hydrolyzing enzymes. The central region is known as (α/β)_8_ barrel domain (residues 129–501); this region of Gt-Mamy displays 44.5% similarity and 14.5% identity to that of Gt-amy. The residues D328, E356 and D424, which are invariably conserved among all the members of GH-13 family, are present in this region. The C-domain comprised the amino acid residues 502–588.

### Cloning and expression, and purification and characterization of the recombinant *Gt-Mamy*

**Figure 1:**
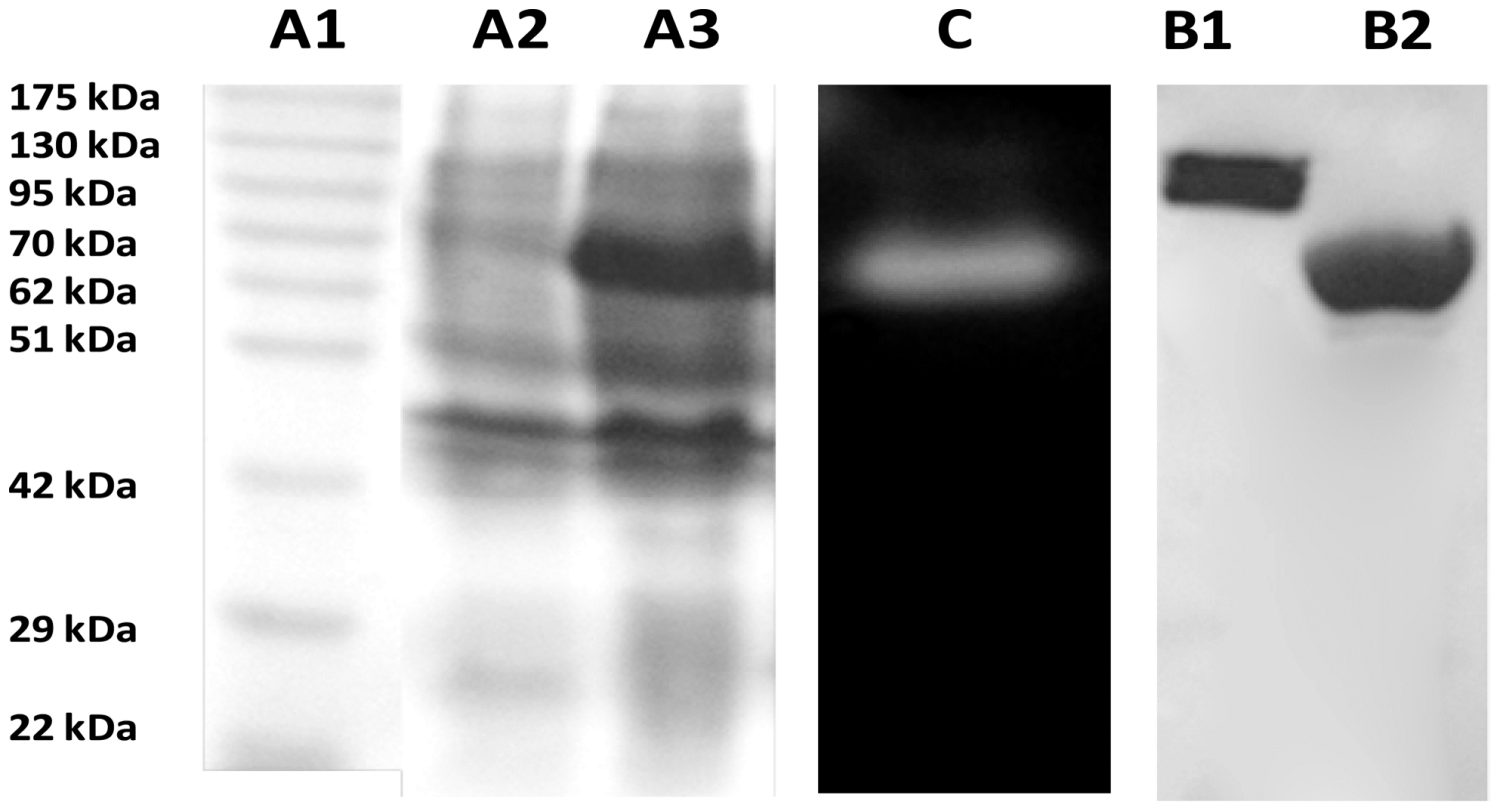
Polyacrylamide gel electrophoresis of the over-expressed and purified Gt-Mamy. Lane A1: molecular weight marker; lane A2: protein profile of uninduced cell lysate; lane A3: protein profile of IPTG-induced cell lysate; lane C: activity staining of the active eluent; lane B1: gel-filtration purified dimeric Gt-Mamy; lane B2: gel-filtration purified monomeric Gt-Mamy.

When the 1767 bp *Gt-Mamy* gene was amplified and cloned into pCOLD™ I vector and pCOLD-*gt-Mamy* construct was transformed into *E. coli* BL21 (DE3), Gt-Mamy was expressed at 15°C using 0.5 mM IPTG. Figure 1 presents the over expression of Gt-Mamy on SDS-PAGE. The recombinant strain produces 254±1.4 U of Gt-Mamy mg^−1^ total intracellular protein in the recombinant *E. coli*.

The purified protein forms one and two protein bands (72.5 and 145 kDa) on 12% SDS-PAGE under denatured and non-denatured conditions, respectively. The two monomeric and dimeric forms have been separated by the Sephacryl™ S-200 gel-filtration column, and their molecular masses have been confirmed to be 72.5±0.7 kDa and 145±1.2 kDa, respectively (Figure 1).

**Figure 2:**
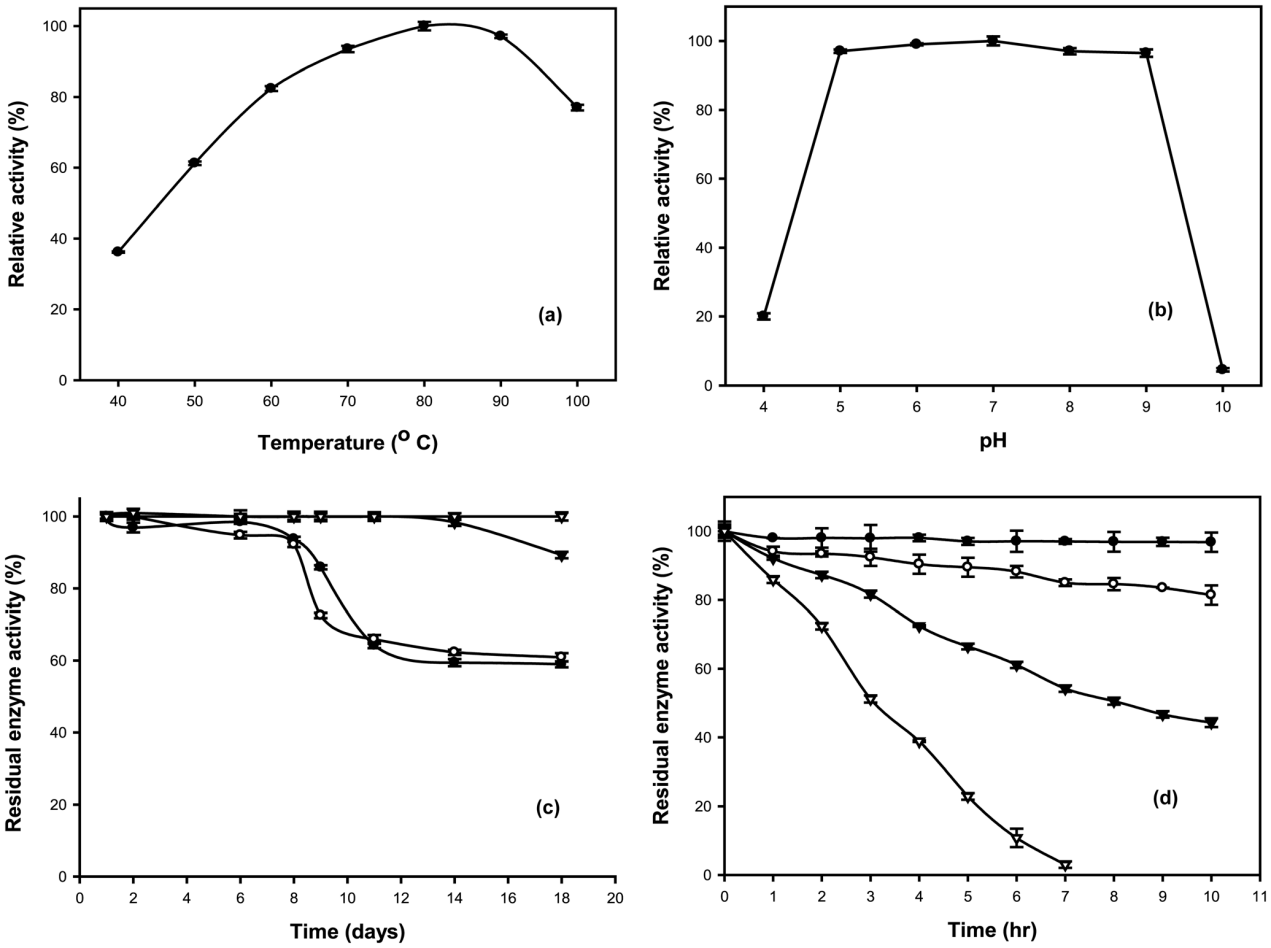
Biochemical properties of Gt-Mamy. (a) Effect of temperature on Gt-Mamy activity; (b) Effect of pH on Gt-Mamy activity; (c) pH stability of Gt-Mamy at room temperature at pH 5 (closed circles), 6 (open circles), 7 (closed triangles) and 8 (open triangles); (d) Thermostability of Gt-Mamy at 70°C (closed circles), 80°C (open circles), 90°C (closed triangles), 100°C (open triangles).

Recombinant Gt-Mamy is optimally active at 80°C, and it declined at 90 and 100°C (Figure 2a). The enzyme is active over a wide range of pH between 5 and 9 (Figure 2b), but the activity drastically declined at pH 4 and 10. The recombinant Gt-Mamy is, therefore, extremely thermostable and pH stable. The pH stability profile of monomeric Gt-Mamy is shown in Figure 2c. There is no loss in the activity of the enzyme till 14 days of incubation at pH 7 and 8, but it decreased drastically after 8 days at pH 5 and 6. The high thermostability of Gt-Mamy over a wide pH range is an advantage for its applicability in starch saccharification, formation of prebiotics via transglycosylation, baking and detergent industries.

Among the metal ions tested, Co^2+^, Mn^2+^, Al^3+^ and Na^+^ stimulated the Gt-Mamy activity (Table 1), while K^+^, Ni^2+^, Ca^2+^ and Ba^2+^ did not exert any observable effect on the Gt-Mamy. The activity was slightly inhibited by Mg^2+^, while Cu^2+^ and Hg^2+^ strongly inhibited (Table 1). Strong inhibition by Cu^2+^ and Hg^2+^ indicates the role of thiol/carboxyl group and indole-containing amino acid residues, respectively, in the enzyme function and catalysis [12]. EGTA [ethylene glycol-bis(2-aminoethylether)-*N,N,N′,N′*-tetraacetic acid] and EDTA [ethylenedia-mine tetraacetic acid] had no observable effect on the activity, suggesting that the enzyme does not require Ca^2+^ or any other metal ion for its activity (Table 1). Slight inhibition by diethylpyro- carbonate (DEPC) and Woodward's reagent K (WRK) indicated the role of histidine and acidic amino acids, respectively in the catalysis and/or substrate binding [13], [14]. Complete inhibition of Gt-Mamy activity by N-bromosuccinamide (NBS) signifies the possible role of tryptophan in maintaining the secondary structure [12].

**Table 1:** Influence of modulators on Gt-Mamy activity.

Modulator/Additive		Relative enzyme activity (%)	
Control	100±1.5		
**Metal ion**	**1 mM**	**5 mM**	**10 mM**
Mg^2+^	84.21±1.0	87.7±1.6	85.2±1.4
Co^2+^	131.2±2.0	113.6±1.2	105.7±1.4
K^+^	102.4±0.5	111.2±1.2	106.3±1.3
Mn^2+^	145.1±1.4	148.1±0.4	142.1±0.8
Ni^2+^	103.6±0.7	104.8±0.5	104.2±0.6
Cu^2+^	0	0	0
Ca^2+^	98.7±1.2	99.0±1.6	98.2±0.7
Ba^2+^	100.2±1.8	100.2±1.3	100.2±0.9
Al^3+^	117.4±0.5	113.6±0.7	108.2±1.2
Na^+^	123.0±1.8	122.4±0.5	123.0±1.8
Hg^2+^	0	0	0
**Inhibitor**	**1 mM**	**5 mM**	**10 mM**
β-ME^1^	73.2±1.1	71.3±0.6	69.9±1.0
PMSF^1^	78.3±1.4	72.4±0.7	62.1±0.8
EGTA^1^	99.4±0.7	98.2±0.7	97.1±1.1
EDTA^1^	99.3±0.5	98.7±1.2	98.4±1.5
NBS^1^	0	0	0
DEPC^1^	91.5±1.1	66.5±0.9	24.5±0.7
WRK^1^	94.5±1.2	79.4±1.1	72.4±0.9

Note: Results are shown as mean ± standard deviation with n = 6.

1 β-ME, β-mercaptoethanol; PMSF, phenyl methyl sulfonyl fluoride; EGTA, ethylene glycol tetra acetic acid; EDTA ethylenediamine tetra acetic acid; NBS, N-bromosuccinamide; DEPC, diethyl pyrocarbonate; WRK, woodword's reagent K.

Gt-Mamy displays less hydrolytic action on raw starches than on soluble starch and that of α-amylase Gt-amy [12]. The enzyme hydrolyzed soluble potato, corn, water chestnut, tapioca, rice, wheat, sago, oat and gram starches to a varied extent (Table 2), but failed to hydrolyze pullulan. Other substrates such as α- cyclodextrins, β-cyclodextrins, amylose and amylopectin were hydrolyzed to a varied extent.

**Table 2:** Action of Gt-Mamy on raw-starches.

Substrate	Relative activity (%)
Corn	55.5±1.3
Water chestnut	79.7±0.6
Tapioca	73.8±0.9
Rice	72.3±1.5
Wheat	61.1±0.5
Sago	82.3±1.1
Oat	48.6±1.0
Gram	71.1±1.4
Pullulan	0.0
Soluble potato (Sigma)	100.0±1.2

Note: Results are shown as means ± standard deviation with n = 6.

**Figure 3:**
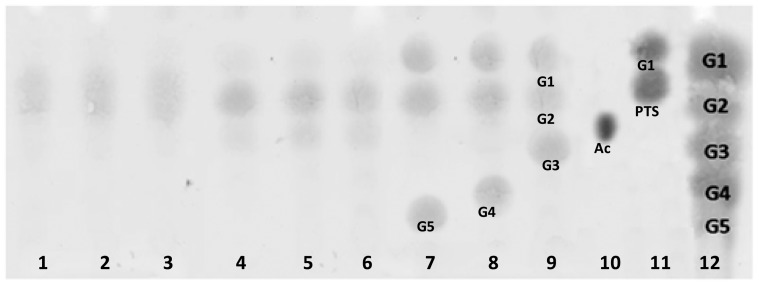
Thin-layer chromatographic analyses of hydrolytic products of substrates. [Starch (lane 1), amylose (lane 2), amylopectin (lane 3), α-cyclodextrin (lane 4), β-cyclodextrin (lane 5), γ-cyclodextrin (lane 6), maltopentaose (lane 7), maltotetraose (lane 8), maltotriose (lane 9), Acarbose (lane 10), Acarbose hydrolysis products, G1 and PTS (pseudotrisaccharide) [Lane 11]; G1, G2, G3, G4, G5, Ac and PTS represents glucose, maltose, maltotriose, maltotetraose, maltopentaoase, acarbose and pseudotrisaccharide, respectively].

Maltogenic amylases are distinguished from the typical α-amylases in the hydrolysis of cyclodextrins and acarbose. TLC analyses of the end-products (Figure 3) showed that the enzyme liberates maltose as the major end product on hydrolyzing starch, amylose and amylopectin (Lanes 1, 2, and 3). The hydrolysis of α-, β-, and γ-cyclodextrins led to the release of maltose and glucose as the major end-products, a property typical of maltogenic amylases [13]. Gt-Mamy hydrolyzes acarbose, a well-known inhibitor of α-amylases, to glucose and pseu-dotrisaccharide (PTS), and G5/G4/G3 to G2 and G1, suggesting that G3 is the smallest oligosaccharide that can be hydrolyzed by Gt-Mamy. Similar observations have been recorded for those from *Thermophilus pendans* and *Thermoplasma volcanicum* GSS1 [2], [8].

### Temperature and pH inactivation of *Gt-Mamy*

The thermal inactivation of Gt-Mamy follows first-order kinetics, and the inactivation rate constant (K_d_) was calculated from the linear regression analysis. Thermal denaturation at elevated temperature is evident by the increase in K_d_ values and a sharp decline in T_1/2_ values with increase in temperature (Table 3). The T_1/2_ values of Gt-Mamy at 80, 90 and 100°C are 35.7, 8.2 and 3.12 h, respectively. The decrease in the T_1/2_ value with increase in temperature occurs as a result of irreversible thermal inactivation of the enzyme due to heat exposure.

**Table 3:** Thermodynamic parameters of Gt-Mamy inactivation.

		80°C	90°C	100°C
**Monomeric Gt-Mamy**	**K_d_ (h^−1^)**	0.01939	0.08458	0.22216
	**T_1/2_ (h)**	35.75	8.20	3.12
	**ΔG (KJ mol^−1^)**	98.52	96.95	96.71
	**ΔS (KJ mol^−1^K^−1^)**	0.091	0.092	0.091
	**ΔH (KJ mol^−1^)**	130.76	130.68	130.60
	**E_a_ (KJ mol^−1^)**	133.69		
**Dimeric Gt-Mamy**	**K_d_ (h^−1^)**	0.01245	0.05571	0.19342
	**T_1/2_ (h)**	55.67	12.63	3.58
	**ΔG (KJ mol^−1^)**	99.82	98.21	97.14
	**ΔS (KJ mol^−1^K^−1^)**	0.134	0.135	0.134
	**ΔH (KJ mol^−1^)**	147.30	147.22	147.13
	**E_a_ (KJ mol^−1^)**	150.23		
**Gt-MamyT**	**K_d_ (h^−1^)**	0.034	0.0926	0.365
	**T_1/2_ (h)**	20.39	4.94	1.90
	**ΔG (KJ mol^−1^)**	96.87	96.68	95.17
	**ΔS (KJ mol^−1^K^−1^)**	0.084	0.082	0.082
	**ΔH (KJ mol^−1^)**	126.76	126.68	126.60
	**E_a_ (KJ mol^−1^)**	129.69		

Dimeric Gt-Mamy exhibits a markedly higher thermostability, and hence, displays lower thermal inactivation as compared to the monomeric form (Table 3). The T_1/2_ values of the dimeric enzyme at 80, 90 and 100°C are 55.7, 12.63 and 3.58 h, respectively. High T_1/2_ and lower K_d_ values of dimeric Gt-Mamy in comparison with the monomeric enzyme indicates thermal stabilization as a result of conformational stabilization and lesser thermal inactivation. Higher thermostability of dimer has also been indicated by the higher E_a_ value (energy required for the thermal denaturation process) for dimer (150.23 KJ mol^−1^) than the monomer (133.69 KJ mol^−1^).

### Conformational stabilization of *Gt-Mamy* as a function of temperature and pH

**Figure 4:**
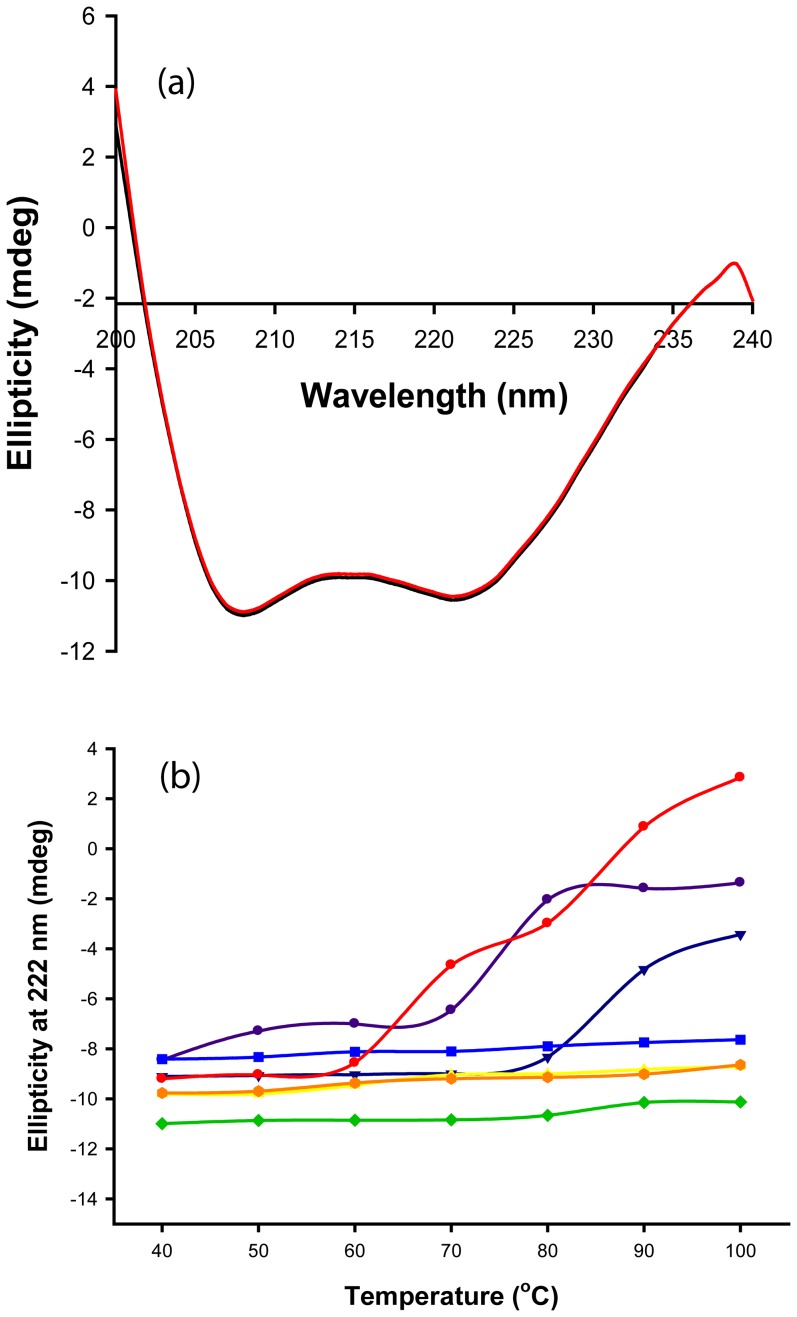
Far-UV CD spectra of Gt-Mamy. (a) Far-UV CD spectra of monomeric (black) and dimeric (red) Gt-Mamy at 80°C and pH 7; (b) Thermal denaturation of dimeric Gt-Mamy at 222 nm at pH 4, pH 5, pH 6, pH 7, pH 8, pH 9 and pH 10 (denoted by purple circle, blue triangle, red circle, blue square, green diamond, yellow triangle and orange circle, respectively). Lines represent the smoothed average of six spectra from which a buffer background has been subtracted.

As visualized from figure 4a, far-UV CD spectra display large CD bands with negative ellipticity at 208 and 222 nm, a distinct property of α-helix rich proteins. Deconvolution of the CD spectra for the secondary structure estimation revealed that the enzyme has α-helix, β-sheet and random coil contents of 67.4, 9.2 and 23.4%, respectively.

Thermal and pH stability of Gt-Mamy was also explained by studying far-UV CD spectra. The negative signal at 222 nm (Figure 4b) remains unchanged in the pH range 6–10 at 40–80°C. There is a slight increase in the ellipticity values at 90 and 100°C, which indicate minor structural changes or shifts. As the deconvoluted CD spectra under these conditions do not show any major change in the α-helix and β-sheet content, this confirms the conformational stability of Gt-Mamy at high pH and temperatures. Sharp increase in the ellipticity values towards zero at pH values 4, 5 and 10, with rise in temperature to 90 and 100°C indicates structural transition of the α-helices to denatured state (Table 4, Figure 4b). The comparison of CD spectra at pH 4, 5 and 10 indicated that pH shift to extremes at lower temperatures (40 to 70°C) does not lead to a major alteration in the enzyme structure. Major structural transition of α-helix content of the enzyme begins as the temperature increases to 80°C, and further, at pH 4 (Table 4). At pH 10, this transition initiates at a much lower temperature. These results confirmed that neither pH nor temperature alone is sufficient for the alteration of its structural conformation. Simultaneous the presence of extreme pH and temperature is necessary for complete denaturation of the enzyme, which confirms its high thermostability as well as pH stability.

**Table 4:** α-Helix, β-sheets and random coils content in the secondary structure of Gt-Mamy.

Temperature	α-helix (%)			β-sheets (%)			Random coils (%)		
	pH 4	pH 5	pH 10	pH 4	pH 5	pH 10	pH 4	pH 5	pH 10
**40°C**	62.1	66.2	66.7	10.0	9.4	9.0	27.9	24.4	24.3
**50°C**	58.2	67.1	66.6	10.2	8.9	9.1	31.6	23.9	24.3
**60°C**	52.5	66.1	66.6	10.6	9.4	8.7	36.9	24.6	24.7
**70°C**	50.2	66.3	46.7	11.7	9.2	14.0	38.1	24.5	39.3
**80°C**	40.2	63.7	32.9	15.4	10.3	20.5	44.4	25.9	46.6
**90°C**	9.7	19.2	7.34	28.7	27.3	29.9	61.6	53.5	62.7
**100°C**	2.24	9.32	1.23	32.8	39.7	35.5	64.9	51.0	63.3

### Thermo-adaptation of *Gt-Mamy*

The dimeric Gt-Mamy is more thermostable than the monomer. Moreover, the sequence analysis of Gt-Mamy suggested a high degree of similarity to the currently known bacterial cyclodextrin hydrolyzing enzymes (88–93%), but it is not clear as to why Gt-Mamy is extremely thermostable, while others are not. The thermodynamics of thermal inactivation of monomeric and dimeric forms of Gt-Mamy have, therefore, been studied by applying the first order kinetics to thermal inactivation data, to assess its thermo-adaptation. The energy required for the process is expressed as ΔH. Opening of the enzyme structure is accompanied by the increase in the disorder of the enzyme structure (ΔS). From the data in table 3, it is clear that there is an insignificant change in the values of ΔH and ΔS with increase in temperature, while ΔG increased. Positive ΔG values for the thermal inactivation of Gt-Mamy indicated non-spontaneity of the process of thermal inactivation, and the enzyme to be thermostable. The changes in ΔH and ΔS become significant when thermal inactivation is related to dimerization. As shown in table 3, values of ΔG, ΔH and ΔS increased upon dimerization.

As ΔH is a measure of enzyme inactivation, higher ΔH signifies greater numbers of non-covalent bonds that are present in the enzyme structure, which need to be broken for thermal inactivation. Increase in ΔH as a result of dimerization makes overall ΔG of the dimer greater than the monomer, making it more stable to thermal denaturation, and hence, greater thermal stability than its monomeric counterpart. Thermostability of an enzyme is a balance of stabilizing and destabilizing forces, which is affected by several factors like number of non-covalent bonds in the enzyme structure, overall hydrophobicity of the enzyme and others [15]. Dimerization causes slight increase in the ΔS of the Gt-Mamy. The slight increase in the ΔS is nullified by a large increase in ΔH as a result of dimerization. In summary, ΔH contributes to the thermostability of the enzyme upon dimerization, making this process enthalpy-driven.

### Kinetics of substrate hydrolysis

Monomeric Gt-Mamy shows higher affinity towards starch as compared to cyclodextrins. Dimerization modulates the affinity of enzyme towards cyclodextrins as can be seen from the K_m_ values, which decrease from 2.01 and 1.87 mg mL^−1^ (monomer) to 0.2 and 0.61 mg mL^−1^ (dimer) for α- and β-cyclodextrin, respectively. The substrate hydrolysis efficiency can be calculated as V_max_/K_m_ ratio [16]. There is 139- and 17- fold increase in the substrate hydrolysis efficiency of Gt-Mamy for α- and β-cyclodextrins, respectively upon dimerization (Table 5). The preference for oligomeric state to bind and hydrolyze cyclodextrins has been described for maltogenic amylases from *Thermoplasma volcanicum* GSS1 and *Thermus* sp. [8], [17].

**Table 5:** Kinetic parameters of monomeric (M) and dimeric (D) Gt-Mamy.

Substrate	K_m_(mg mL^−1^)		V_max_(µmol mg^−1^ min^−1^)		k_cat_(min^−1^)		V_max_/K_m_	
	M	D	M	D	M	D	M	D
**α-cyclodextrin**	2.01	0.20	83.3	1151.2	6.04	166.8	41.4	5756
**β-cyclodextrin**	1.87	0.61	101.5	548.9	7.36	79.5	54.3	899.8
**Soluble starch**	1.40	8.62	166.7	16.2	12.1	2.3	119.1	1.9
**Amylose**	1.34	8.69	174.6	17.7	12.7	2.6	130.3	2.0
**Amylopectin**	2.42	11.32	141.7	15.8	10.3	2.3	48.4	1.4

The hydrolysis efficiency for starch, amylose and amylopectin decreases by a factor of 63, 65 and 35, respectively (Table 5). Sequence comparison of Gt-Mamy and ThMA (1SMA) showed the presence of I431, a bulky amino acid, instead of V431 in ThMA [18] at the substrate entry site. This substitution might explain a decrease in the substrate-binding affinity, turnover number (k_cat_) as well as substrate hydrolysis efficiency (V_max_/K_m_) of Gt-Mamy for starch, amylose and amylopectin upon dimerization. These observations clearly indicate that there is a switch in Gt-Mamy activity from starch hydrolysis to cyclodextrin hydrolysis upon dimerization.

### N-terminal truncation of *Gt-Mamy*

The comparison of amino acid sequence of Gt-Mamy and Gt-amy (α-amylase) reveals the presence of an extra N-terminal 128 amino acids (N-domain) in Gt-Mamy. To determine the impact of N-terminus on the dimeric state of Gt-Mamy, the first 128 amino acids corresponding to the N-domain were deleted. The truncated Gt-Mamy is (designated as Gt-MamyT) was overexpressed and purified, with a molecular mass of ∼59 kDa, which confirms that the truncation leads to monomerization of this enzyme. Similarly N-domain has been shown to play a role in the oligomerization of other cyclodextrin hydrolyzing enzymes [19].

The biochemical characterization of Gt-MamyT suggested that its temperature optima and pH optima are identical to those of Gt-Mamy. Modulators also exhibit similar effects on Gt-MamyT, as reported by Li et al. [1] for the multifunctional amylase of *Bacillus* sp. ZW25311. However, there is a 16- and 3.5- fold reduction in the affinity of Gt-MamyT to α- and β-cyclodextrins (Table 6) as compared to the dimeric form, an effect similar to that of the monomer. There is also a decrease in α- and β-cyclodextrin hydrolysis efficiency of Gt-MamyT by a factor of 232 and 19, respectively, to that of the native dimer. Decrease in the substrate-binding affinity and substrate hydrolytic efficiency of Gt-MamyT towards α- and β-cyclodextrins as compared to that of the native dimer also suggests a structural modification in the enzyme active site. This further suggests the role of N- domain in the modulation of substrate specificity of Gt-MamyT for cyclodextrins.

**Table 6:** Kinetic parameters of truncated and mutant Gt-Mamy.

Substrate	K_m_(mg mL^−1^)			V_max_(µmol mg^−1^ min^−1^)			k_cat_(min^−1^)			V_max_/K_m_		
	Δ128	D109E	D109A	Δ128	D109E	D109A	Δ128	D109E	D109A	Δ128	D109E	D109A
**α-cyclodextrin**	3.21	0.21	0.94	79.6	1149.5	314.5	5.77	83.33	45.29	24.8	5473.8	334.6
**β-cyclodextrin**	2.12	0.60	1.23	99.7	550.3	203.6	7.22	39.89	29.32	47.0	917.2	165.5
**Soluble starch**	1.68	8.21	3.43	162.5	17.4	51.23	11.8	1.26	7.38	96.7	2.1	14.9
**Amylose**	1.57	8.13	3.18	172.4	18.5	54.32	12.5	1.34	7.82	109.8	2.3	17.1
**Amylopectin**	2.94	21.43	7.54	38.6	4.12	42.8	2.8	0.29	6.16	13.1	0.19	5.7

Indirect or direct influence of N- domain in the alteration of thermostability of Gt-MamyT has been elucidated by monitoring its thermal denaturation pattern. The truncated enzyme displays a decreased thermostability as compared to both monomeric and dimeric Gt-Mamy (Table 3), as evident from the lower T_1/2_ and greater K_d_ values at elevated temperatures. The decreased thermal stability also explains the lower E_a_ value of Gt-MamyT. These observations further validate N-terminus to play a role in homodimer formation and thermosta-bilization of Gt-Mamy. When thermodynamic parameters of Gt-MamyT inactivation were calculated, a decrease in ΔH and ΔG values was recorded (Table 3); this explains the decreased thermostability of the truncated enzyme due to the loss of dimerization, which is an indirect effect like that of the monomeric Gt-Mamy.

### Molecular docking of β-cyclodextrin to mal_amy model

Gapped-BLAST analysis revealed 95, 87, 53 and 46% structural identities of the target sequence with maltogenic amylase of *Thermus* sp. (1SMA_B), neopullulanase of *G. stearother-mophilus* (1J0H_B) and cyclomaltodextrinase of *Bacillus* sp. (1EA9_D) and α-amylase of *Thermoactinomyces vulgaris* (1JI2_A), respectively. The homology model of Gt-Mamy was built as a homodimer using 1SMA_B as a template. Figure S3a clearly shows that the N-terminal domain of chain A and B of Gt-Mamy participate in the dimerization of the Gt-Mamy. The N-terminal domain of chain A covers a part of (α/β)_8_ barrel of the chain B and vice-versa, to form two narrow and deep catalytic clefts (Figure S3f), which closely resembles the active-site cleft (∼17 Å long, 8 Å wide and 18 Å deep) of ThMA (*Thermus* Maltogenic amylase) [20] rather than TVA II (*Thermoactinomyces vulgaris* amylase II) [21]. This narrow and deep groove (Figure S3g,h) is particularly different from the wide and shallow active site cleft of other α-amylases and maltogenic amylase of *Thermoactinomyces vulgaris*.

The molecular docking of β-cyclodextrin to Gt-Mamy model was carried out to interpret the observations explained earlier, and to locate N-terminal amino acid residues responsible for binding to cyclodextrin, and modulating its substrate-binding affinity and thermostability via dimerization. The free energy of β-cyclodextrin binding to dimeric Gt-Mamy is more negative (−4.51 kcal mol^−1^) than the monomeric Gt-Mamy (−2.05 kcal mol^−1^). Furthermore, the frequency of docking runs resulting in the same geometry is more (80%) for dimer than the monomeric form (30%).

Higher negative free energy of binding and frequency, obtained after docking of β-cyclo-dextrin to dimeric Gt-Mamy than the monomer, indicates that the docking of β-cyclo-dextrin to dimeric Gt-Mamy doesn't require reorientation of the protein chains and change in the torsion angle of the β-cyclodextrin molecule, ensuring that binding of β-cyclodextrin to dimer is reliable and specific as compared to the monomer. Amino acid residues involved in the binding of β-cyc-lodextrin to the dimeric Gt-Mamy are Y45, W47, D109, T111 of chain A and H205, F289, V292, M295, D328, E357, T423, D424, I431 of chain B. While residues Y45 and W47 make hydrophobic interaction with the inner ring of β-cyclodextrin, T111 and E357 form polar interaction. Amino acid residues involved in the binding of β-cyclodextrin to the monomeric Gt-Mamy are Y45, W47, D110, T111 and A112.

**Figure 5:**
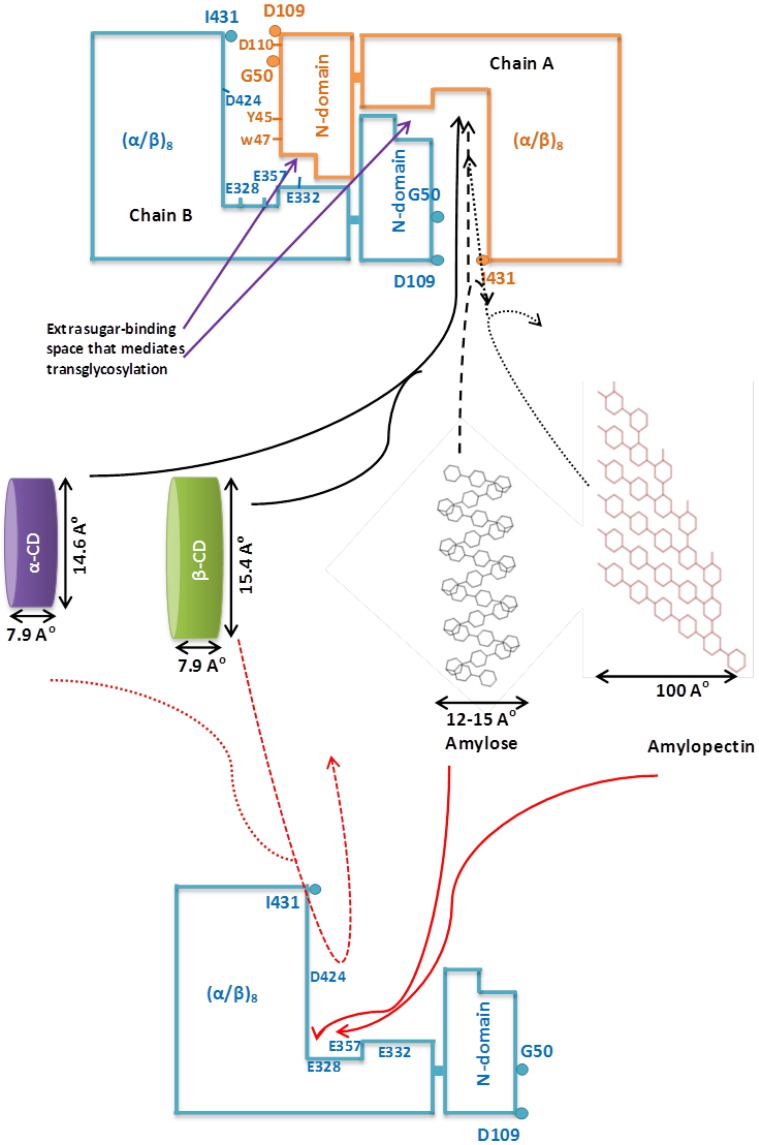
Substrate specificity of Gt-Mamy. Two chains A and B of dimeric Gt-Mamy are shown. Residues at the substrate entry site are shown as circles. Important residues responsible for β-cyclodextrin binding in chain B and A are shown as amino acid symbols followed by position. Entry and exit of high affinity substrates are shown by solid arrows. Entry and exit of low affinity substrates are shown by dotted arrows (Enzyme cartoon of the dimer is modified from Park et al. (2007)].

The docking model explains that β-cyclodextrin interacts with some residues from the (α/β)_8_ barrel of one chain and N-terminal domain of the second chain, and vice-versa to form two substrate binding clefts as a result of its dimerization. Catalytic residues D424, D328 and E357, which come in the proximity of β-cyclodextrin via dimerization, form hydrogen bond, polar interactions and polar interactions with β-cyclodextrin, respectively. Consequently, it forms the deep and narrow cyclodextrin-binding grove into which β-cyclodextrin molecule fits through the stable interactions (Figure S3d, figure S3e, figure 5). Increased affinity of the dimer to cyclode-xtrins results from the formation of a deep and narrow cyclodextrin-binding catalytic pocket in comparison with the wide and shallow active site in the monomer. This deep and narrow catalytic pocket permits easy access of flat and thin cyclodextrins [14.6/15.4 Å (α/β-cyclodextrin) high and 7.9 Å wide] with more stabilizing interactions, and thus, causes steric hindrance for the entry of coiled helices of amylose (12–15 Å wide), amylopectin (100 Å wide) and starch, as reported for a few cyclodextrin-hydrolyzing enzymes (Figure 5) [17], [22]. The wide and shallow active site in the monomer eases the entry of larger molecules of amylose and amylopectin and makes more stable interactions than cyclodextrins that fit loosely; this explains higher affinity of monomeric enzyme for starch, amylose and amylopectin. The preferential affinity of Gt-Mamy to cyclodextrins is, therefore, related to its structural arrangement, homo-dimer formation.

### Characterization of D109 mutants

According to docking results, an N-terminal amino acid residue out of four that mediates binding to β-cyclodextrin is D109. In a dimeric Gt-Mamy, I431 of chain A interacts with D109 and G50 of chain B as shown in the figure 5, forming the substrate entry site. Site directed mutagenesis was, therefore, carried out at this position to understand the role of D109 on the substrate affinity and dimerization state of Gt-Mamy. The mutated forms of Gt-Mamy (D109E and D109A) were generated. SDS-PAGE as well as zymogram analysis of the D109E mutein revealed the occurrence of dimeric form. Moreover, D109E mutein does not show any effect on the binding affinity and substrate hydrolytic efficiency towards α- and β-cyclodextrins (Table 6). A strong decline in the affinity and substrate hydrolytic efficiency of the mutant enzyme towards amylopectin has been observed, as evident from the K_m_ value of 21.43 mg mL^−1^ and V_max_/K_m_ value of 0.19, which are 1.9 and 7.3 times less than the dimeric wild-type Gt-Mamy, respectively. Site directed mutagenesis at the substrate entry site resulted in 2.3-fold decrease in the affinity of ThMA to amylopectin [18].

In contrast, D109A mutant shows increased affinity towards amylose, amylopectin and starch, and a decreased affinity towards α- and β-cyclodextrins. As summarised in the Table 6, there is 2.5, 2.7 and 1.5 fold increase in its affinity towards starch, amylose and amylopectin, and 4.7 and 2 fold decrease in the affinity of mutant enzyme to α- and β-cyclodextrins, respectively. Increased affinity of D109A towards amylose, amylopectin and starch, and a slight decreased affinity towards α- and β-cyclodextrins, might have occurred as a consequence of destabilization effect of this mutation on the enzyme structure. Due to this destabilization effect, the mutant enzyme dissociates into the monomeric form and is present in monomer-dimer equilibrium in the solution, and therefore, K_m_ values of this mutein to different substrates lie in between the values for monomer and dimer, and hence, the substrate binding affinities too.

D109E mutant enzyme shows insignificant changes in T_1/2_ at the elevated temperatures, while D109A exhibits decreased T_1/2_ values. The T_1/2_ values of D109A at 80, 90 and 100°C are 44.6, 8.9 and 2.82 h, respectively. Decline in the T_1/2_ values, with concomitant decrease in the E_a_ required for its thermal denaturation to 141.4 KJ mol^−1^, explains decreased thermostability as a result of partial loss of dimerization state of the enzyme and its dissociation to the monomeric form. These results indicate the role of D109 in the dimerization of Gt-Mamy and its indirect role in the modulation of substrate preferences and thermostability.

### Effect on N-terminal truncation on the transglycosylation of sugars

**Figure 6:**
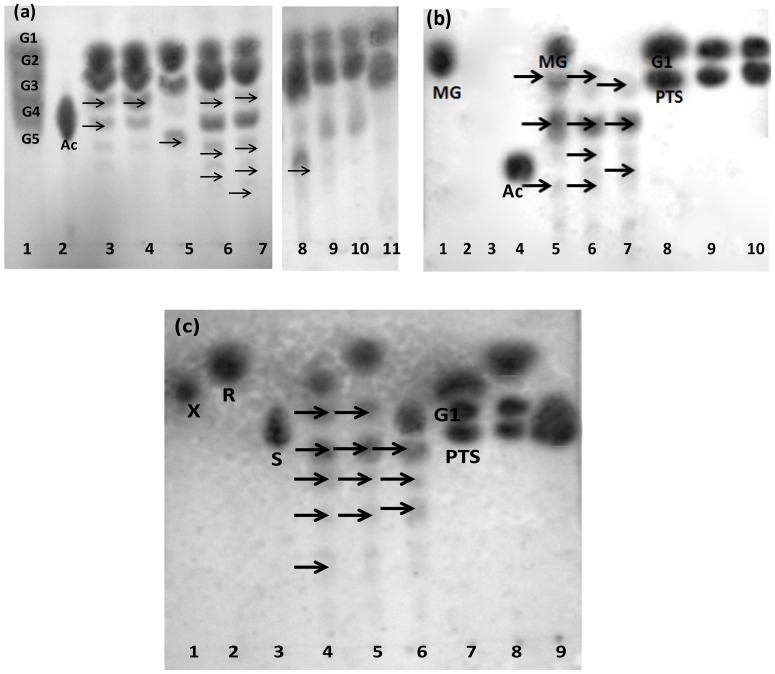
Transglycosylation activity of Gt-Mamy. (a) Transglycosylation of hydrolytic products of G4, G5 and acarbose onto G1/G2 (Lanes 1,2,3,4,5,6,7,8,9,10,11 indicate standards, Ac, G1+G5+E, G1+G4+E, G1+Ac+E, G2+G5+E, G2+G4+E, G2+Ac+E, G1+G4+EΔ, G1+G5+EΔ, G1+Ac+EΔ, respectively); (b) Transglycosylation of hydrolytic products acarbose onto methyl-α-D-glucopyranoside (MG), mannitol (ML)and xylitol (XL) [Lanes 1,2,3,4,5,6,7,8,9,10 indicate MG, ML, XL, Ac, Ac+MG+E, Ac+ML+E, Ac+XL+E, Ac+MG+EΔ, Ac+ML+EΔ, Ac+XL+EΔ, respectively; (c) Transglycosylation of hydrolytic products acarbose onto xylose (X), rhamnose (X) and sucrose (S) [Lanes 1,2,3,4,5,6,7,8,9,10 indicate X, R, S, Ac+X+E, Ac+R+E, Ac+S+E, Ac+X+EΔ, Ac+R+EΔ, Ac+S+EΔ] (Abbreviations: G1, G2, G3, G4, G5, Ac and PTS stands for glucose, maltose, maltotriose, maltotetraose, maltopentaose, acarbose and pseudotrisaccharide, respectively; arrows indicate transglycosylated products).

Dimeric Gt-Mamy is able to transglycosylate acarbose, G4 and G5 onto various acceptor molecules. Figure 6 presents the TLC analyses of transglycosylated products of dimeric Gt-Mamy and Gt-MamyT. Gt-Mamy simultaneously hydrolyzes G4, G5 and acarbose and transglycosylates its hydrolytic products onto acceptor molecules G1 and G2, or vice-versa (Figure 6a, lanes 3–8). In contrast, Gt-MamyT hydrolyzes G4, G5 to G2 and G1, and acarbose to G1 and PTS, but fails to transglycosylate its hydrolytic products to the acceptor molecules (Figure 6a, lanes 9–11). Besides G2 and G1, Gt-Mamy also transglycosylates the products of acarbose hydrolysis onto other acceptor sugars and sugar alcohols like methyl-α-D-glucopyranoside (MG), mannitol, xylitol, xylose, rhamnose and sucrose. Figure 6b and 6c displays transglycosylation of acarbose hydrolytic products to methyl-α-D-glucopyranoside (MG), mannitol (ML), xylitol (XL), xylose (X), rhamnose (R) and sucrose (S) by Gt-Mamy (Figure 6b, lanes 5–7), while Gt-MamyT does not exhibit any transglycosylation activity, although it hydrolyzes acarbose to G1 and PTS (Figure 6b, lanes 8–10; Figure 6c, lanes 7–9).

The possible explanation for the absence of transglycosylation activity in Gt-MamyT could be due to the absence of N-terminal domain which does not allow the formation of extra sugar-binding space (Figure S3), and thus, this truncated enzyme cannot accommodate sugar molecules for transglycosylation. N-terminal domain, therefore, plays an indirect role in the transglycosylation activity of Gt-Mamy.

## Conclusions

Bacterial maltogenic amylase with high thermostability and broad pH range for activity is being reported for the first time. The enzyme is distinct from the α-amylase (Gt-amy) of *G. thermoleovorans* reported earlier. The maltogenic amylase has greater affinity for cyclodextrins as compared to starch, and further, hydrolyzes acarbose, an inhibitor of α-amylase. Gt-Mamy forms a homodimer and dimerization mediates enthalpy-driven conformational thermostabilization of the enzyme. N-terminal truncation has indicated that dimerization modulates substrate-binding affinity and transglycosylation activity of the enzyme. Molecular docking of β-cyclodextrin to Gt-Mamy and SDM aided in confirming the role of N-terminal D109 at the substrate entry site in dimer formation.

## Materials and Methods

### Materials

*Escherichia coli* DH5α and *E. coli* BL21 (DE3) (Novagen) were used as the host strains for cloning and expression of maltogenic amylase gene from *Geobacillus thermoleovorans* (MTCC 4220, 16S rDNA sequence accession no. JN871595). Vector pCOLD™ I was procured from Takara Bio Inc., Japan. Primer oligonucleotides and Ni^2+^-NTA agarose resin were purchased from Sigma (St Louis, USA) and Novagen, respectively.

### DNA manipulation

Genomic DNA was extracted according to Bazzicalupo and Fani [23]. A set of internal oligonucleotide primers (Mal_int_F/R, table 7) were designed from the conserved regions of the maltogenic amylases of the known bacterial species. Approximately 50 ng of genomic DNA was used as a template for the amplification of this internal region of the *Gt-Mamy* in a Thermocycler (Bio-Rad, USA) in 50 µl reaction mixture (initial denaturation at 95°C for 5 min followed by 30 cycles of denaturation at 95°C for 50 sec, annealing at 59°C for 30 sec, and extension at 72°C for 1 min, and a final extension of 5 min at 72°C) using Taq DNA polymerase (New England Biolabs, MA). This amplicon was cloned into pGEM®-T easy vector and sequenced at Nucleic Acid Sequencing Facility, University of Delhi South Campus, New Delhi. This amplicon shares significant homology with the α-cyclodextrinase of *G. stearothermophilus* (GenBank ID AB070710.1), α-amylase catalytic region of *Geobacillus* sp. Y412MC52 (GenBank ID CP002442.1) and maltogenic amylase of *Geobacillus* sp. Gh6 (GQ884176.1).

**Table 7:** Sequences of oligonucleotides employed in this investigation.

Primer name	Sequence (5′ to 3′)
Mal_int_F	5′ GCTGGCGTCTTGATGTTGCCAA 3′
Mal_int.R	5′ ATCATGGCTGCCAAGCAA 3′
Mal_GT_F	5′ ATGAGGAAAGAAGCCATCCACC 3′
Mal_.GT_R	5′ TTACCAGCTTTCGACCGCGTA 3′
Mal_GT_Sa_F	5′ CCC**GAGCTC**ATGAGGAAAGAAGCCATCCACC 3′
Mal_GT_Xh_R	5′ CCC**CTCGAG**TTACCAGCTTTCGACCGCGTA 3′
D109E_F	5′ TGAAGCTCCGAGCGACGA**A**ACCGCTTACTACTTTTGC 3′
D109E_R	5′ GCAAAAGTAGTAAGCGGT**T**TCGTCGCTCGGAGCTTCA 3′
D109A_F	5′ ATGAAGCTCCGAGCGACG**CT**ACCGCTTACTACTTTTGC 3′
D109A_R	5′ GCAAAAGTAGTAAGCGGT**AG**CGTCGCTCGGAGCTTCAT 3′

Note: Bold and underlined nucleotide sequence corresponds to the restriction enzyme sites (6 bases) or mutated bases (1 or 2).

The end primers (Mal_GT_F/R, table 7) were designed from the maltogenic amylase sequences and the full-length *Gt*-M*amy* was amplified in a 50 µl reaction mixture (initial denaturation at 95°C for 5 min followed by 30 cycles of denaturation at 95°C for 50 sec, annealing at 64°C for 30 sec and extension at 72°C for 1 min 30 sec, and a final extension of 5 min at 72°C) using Taq DNA polymerase and sequenced by automated sequencing at Nucleic Acid Sequencing Facility at University of Delhi South Campus, New Delhi. The compatible restriction enzyme sites of *SacI* and *Xho*I were created at the end of the Mal.GT.F/R to create Mal_GT_Sa_F/Xh_R (Table 7). The digested PCR amplicon and pCOLD™ I vector were purified using Qiagen PCR purification kit, ligated and transformed into *E. coli* DH5α competent cells. Positive clones were confirmed by colony PCR and double digestion of the construct by *SacI* and *Xho*I, and were sequenced.

### Bioinformatic analysis and homology modeling of *Gt-Mamy*

Multiple sequence alignment (MSA) was carried out using ClustalW program [24]. The template selection for the homology model was carried out based on the analysis of scores, E-values and percent identities of the gapped-BLAST generated sequences that produced significant alignment with the *in silico* translated sequence of Gt-Mamy. Automated homology modelling was carried out using the template using SWISS-MODEL [25], and the model was visualized by the DeepView - Swiss-PdbViewer 4.1.0 [26].

### Molecular docking of β-cyclodextrin to *Gt-Mamy* model

β-cyclodextrin ligand (CID 444041, SID 57280122) was downloaded from the PubChem Compound Database at NCBI (National Center for Biotechnology Information). Docking calculations were carried out using Docking Server [27]. Gasteiger partial charges were added to the ligand atoms. Non-polar hydrogen atoms were merged, and rotatable bonds were defined. Docking calculations were carried out on β-cyclodextrin Mal_amy model. Essential hydrogen atoms, Kollman united atom type charges, and solvation parameters were added with the aid of AutoDock tools [28]. Affinity (grid) maps of 20×20×20 Å grid points and 0.375 Å spacing were generated using the Autogrid program [28]. AutoDock parameter set- and distance-dependent dielectric functions were used in the calculation of the van der Waals and the electrostatic terms, respectively.

Docking simulations were performed using the Lamarckian genetic algorithm (LGA) and the Solis & Wets local search method [29]. Initial position, orientation, and torsions of the ligand molecules were set randomly. Each docking experiment was derived from 10 different runs that were set to terminate after a maximum of 250000 energy evaluations. The population size was set to 150. During the search, a translational step of 0.2 Å, and quaternion and torsion steps of 5 were applied.

### N-terminal truncation of *Gt-Mamy*

To truncate the *Gt-Mamy* gene from the N-terminus, primer Trn_amy_F (5′ CCC**GAGCTC**CC-GGACTGGGTAAAAGACAC 3′) was designed deleting the 128 amino acids from the N-terminus region. This forward primer and Mal_GT_Xh_R was used to amplify the truncated G*t-Mamy* gene (designated as *Gt-MamyT*). This amplicon was digested with SacI and XhoI and cloned in the pCOLD™ I vector as mentioned previously.

### Site-Directed Mutagenesis (SDM)

PCR-based SDM was carried out using the GENEART site directed mutagenesis kit (Invitrogen, Carsband, USA). A set of overlapping oligonucleotide primer pairs with the desired mutations (Table 7) were designed to generate the mutant constructs for D109A and D109E mutants. Approximately 30–40 ng of recombinant vector was used as a template for the amplification of the mutant constructs in a Thermocycler (Bio-Rad, USA) in a 50 µl reaction mixture according to manufacturer's protocol. The plasmid with the mutated gene was transformed into the competent cells of *E. coli* DH5αTM-T1^R^. The plasmid constructs isolated from the transformants were sequenced for the confirmation of the mutated nucleotide(s).

### Expression of the recombinant *Gt-Mamy* and its mutants, and their purification

Plasmid construct was transformed into *E. coli* BL21 (DE3), and the transformed cells were cultured at 37°C for 16–18 h in LB medium supplemented with ampicillin (100 µg mL^−1^). The expression of *Gt-Mamy* gene under the control of *cspA* promoter was performed as described by Qing et al. [30]. The whole-cell protein profiles of the induced and uninduced cultures were analysed on 12% SDS-PAGE. The gel was run at 35 mA constant current till the dye front reaches the end. The recombinant enzyme was purified using Ni^2+^-NTA affinity chromatography under non-denaturing conditions at pH 7.0. Elution was carried out with 150 mM imidazole and analysed on SDS–PAGE as described by Laemmli [31]. Active fractions were dialyzed against 100 mM sodium phosphate buffer (pH 7) and stored at 4°C. The protein concentration was determined according to Lowry et al. [32]. Zymogram analysis was carried out using SDS-PAGE as described earlier [12].

Monomeric and dimeric forms of the purified Gt-Mamy were separated by FPLC (AKTAPrime plus). The Sephacryl™ S-200 high resolution column (Pharmacia) was pre-equilibrated with sodium phosphate buffer (100 mM, pH 7) containing 50 mM NaCl and the purified enzyme containing monomeric and dimeric forms were loaded, and eluted at a flow rate of 0.3 ml min^−1^. Cytochrome c (12.4 kDa), carbonic anhydrase (29 kDa), bovine serum albumin (66 kDa), yeast alcohol dehydrogenase (150 kDa), and sweet potato β- amylase (200 kDa) were used as standards to calculate the molecular mass of the purified enzymes. Molecular mass of the monomeric and dimeric forms of Gt-Mamy were determined using a plot of molecular weight Vs Ve/Vo.

### Amylase assay

To 0.5 mL appropriately diluted enzyme (1.5 U), 0.5 mL 0.5% potato starch or β-cyclodextrin (Sigma, St. Louis, MO) prepared in 100 mM sodium phosphate buffer (pH 7.0) were added, mixed and incubated at 80°C for 10 min. The reducing sugars liberated were determined using 3, 5-dinitrosalicylic acid (DNSA) reagent [33]. One unit of α-amylase is defined as the amount of enzyme that liberates 1 µmol of reducing sugars as maltose per min under the assay conditions.

### Biochemical characterization and enzyme kinetics

The optimum pH for the activity of pure recombinant enzyme was determined by incubating the reaction mixtures at 80°C in buffers of varying pH. The optimum temperature for the enzyme activity was determined by incubating the reaction mixtures (pH 7.0) at 40°C–100°C. The reducing sugars liberated were quantified. Biochemical characterization, end-product analysis and substrate spectrum analysis of Gt-Mamy were carried out at pH 7.0 and 80°C [12]. Soluble potato starch (Sigma, St. Louis, USA) solutions of varying concentrations of starch (0.1%–1.0%) and diluted enzyme (1.5 U) were prepared in 100 mM sodium phosphate buffer (pH 7.0). The enzyme activities determined at 80°C were used in calculating K_m_, V_max_ and k_cat_.

### Kinetics and thermodynamics parameters of enzyme inactivation

Thermal stability was assessed by incubating the enzyme (pH 7.0) at different temperatures (80°C–100°C) followed by assay at the desired intervals. The residual enzyme activities were determined at each temperature. This data was fit into the first-order kinetics and K_d_ (deactivation rate constant), half-life (T_1/2_) and energy required for thermal inactivation (E_a_) of the Gt-Mamy inactivation were calculated [12].

Thermodynamic parameters of thermal inactivation of the Gt-Mamy were calculated by rearranging the Eyring's absolute rate equation from the transition state theory.

Eyring's absolute rate equation,
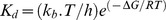
that corresponds to
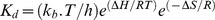
Or
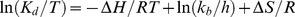
was used to calculate ΔH (change in enthalpy of deactivation of the enzyme), ΔH (entropy of deactivation of the enzyme) and ΔG (free energy of the enzyme deactivation), where h is the

Planck's constant (6.626×10^−34^), k_b_ is the Boltzmann constant (1.381×10^−23^ J K^−1^), R is the gas constant (8.314 J K^−1^) and T is temperature in K.

### Effect of temperature and pH on the secondary structure of Gt-Mamy

The secondary structure of purified Gt-Mamy (0.25 mg mL^−1^) was determined by CD spectroscopy in a JASCO-815 Spectropolarimeter equipped with in-built Peltier controlled thermostat cell holder (CDF-423S). Nitrogen was flushed continuously through the instrument at the rate of 5.0 ml min^−1^, and the path length of cuvette used was 10 mm. The effect of temperature and pH on Gt-Mamy was investigated by performing the scans at different temperatures (30 to 100°C) and different pH (4–11). The changes in the structural conformation were recorded in the wavelength range 200 to 240 nm at a scanning rate of 50 nm min^−1^. Each CD spectrum is an average of six scans, and the data acquisition and analysis were performed on a computer interfaced to the instrument. Data analysis was done by K_2_D_2_ software.

### Transglycosylation of acceptor molecules by *Gt-Mamy* and its mutants

Applicability of Gt-Mamy and Gt-MamyT was studied in the transglycosylation of sugars to various acceptor molecules. 5 mg each of the donor and acceptor sugars were mixed in a 50 µl reaction and incubated at 70°C for 1 h. After 1 h, 5.0 U of the purified enzyme was added to the mixture and the incubation was continued for 24 h. The products were analyzed by thin layer chromatography [12].

### Sequence accession numbers

The *G. thermoleovorans* g*t-Mamy* sequence was submitted to the GenBank database and assigned accession no. JQ999960. The protein ID of the amino acid sequence is AFM43699.

## Supporting Information

Figure S1 Schematic representation of sequencing of full-length *gt-Mamy* gene by primer walking. Full-length gene was sequenced in three parts; Region I was sequenced first and then extended towards both the directions for the sequencing of regions II and III; forward and reverse primers are shown by forward and backward arrows.
(TIF)

Figure S2 Multiple sequence alignment of *in silico* translated Gt-Mamy with the other closely related enzymes [*G. thermoleovorans* CCB_US3_UF5 maltogenic amylase (GenBank ID YP_004981205.1), *Thermus* sp. YBJ-1 α-cyclodextrinase (GenBank ID AAL62457.2), *G. caldoxylosilyticus* maltogenic amylase (GenBank ID ACN79585.1), *G. stearothermophilus* maltogenic amylase (BSMA, GenBank ID AAC46346.1)]. Highlightened regions indicate the seven conserved regions of the α-amylase family. Catalytically active residues are marked by a blue triangle. α-helices and β-strands are indicated by arrows (α-helices by green arrow and β-strands by orange arrows). PDB ID of the template is 1SMA (*Thermus* sp. IM6501 maltogenic amylase). [Note: 1. Abbreviations: α (α-helix), β (β-strand); 2. ‘*’ indicates the residues in the column that are identical in all sequences, ‘:’ indicates conserved substitutions, ‘.’ indicates semi-conserved substitutions]. GenBank IDs for *gt-Mamy* and Gt-Mamy are JQ999960.1 and AFM43699.1, respectively.
(TIF)

Figure S3 Homology modelling and molecular docking. (a) Quaternary structure of Gt-Mamy showing two chains, A and B; (b) Catalytic residues of Gt-Mamy; (c) Active-site cleft of Gt-Mamy; (d) Molecular docking of β-cyclodextrin to Gt-Mamy (residues that show direct interaction with β-cyclodextrin are shown as surface); (e) Docked sites of β-cyclodextrin binding; (f) Dimeric Gt-Mamy with two β-cyclodextrin binding pockets; (g) Close view of β-cyclodextrin binding pocket I; (h) Close view of β-cyclodextrin binding pocket II.
(TIF)
