# Supplementary figures and images for: Dimerization Mediates Thermo-Adaptation, Substrate Affinity and Transglycosylation in a Highly Thermostable Maltogenic Amylase of Geobacillus thermoleovorans
Source: PLoS One. 2013 Sep 19;8(9):e73612. doi: 10.1371/journal.pone.0073612 (PMC3777949; doi:10.1371/journal.pone.0073612)

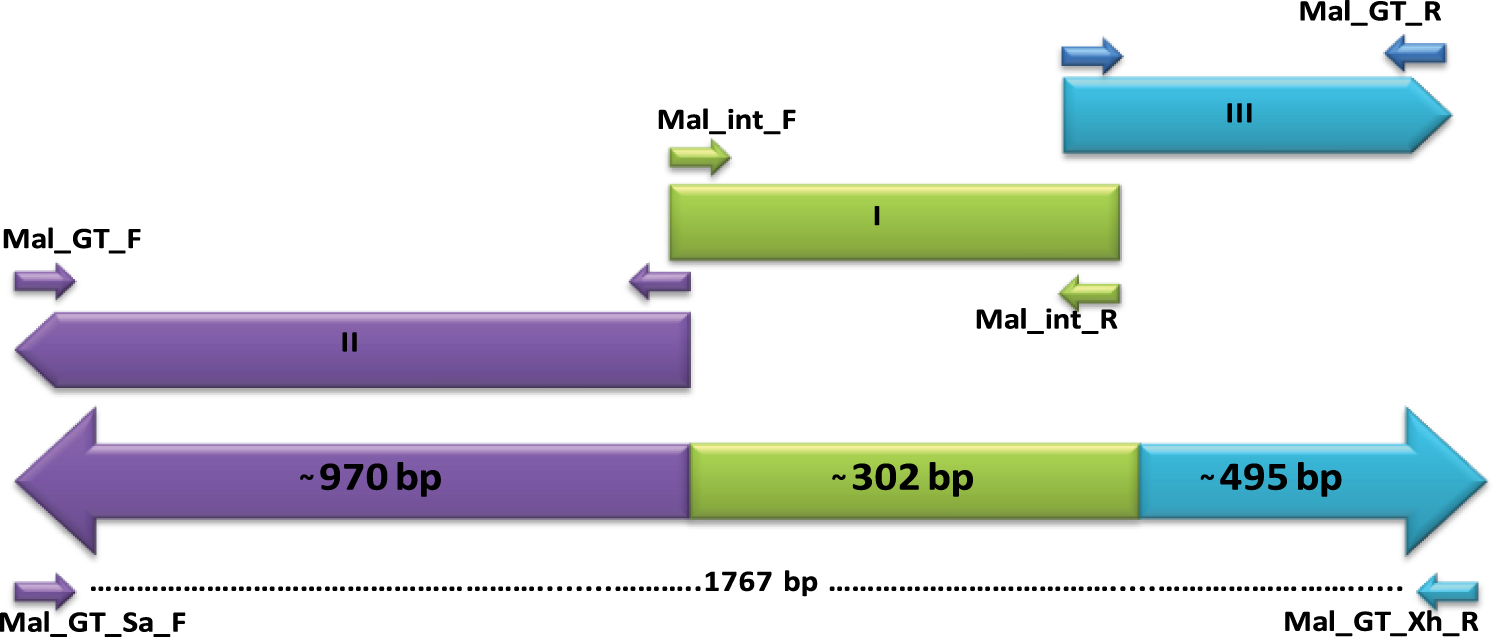

Supplement: Figure S1 — Schematic representation of sequencing of full-length gt-Mamy gene by primer walking. Full-length gene was sequenced in three parts; Region I was sequenced first and then extended towards both the directions for the sequencing of regions II and III; forward and reverse primers are shown by forward and backward arrows. (TIF) [file pone.0073612.s001.tif]

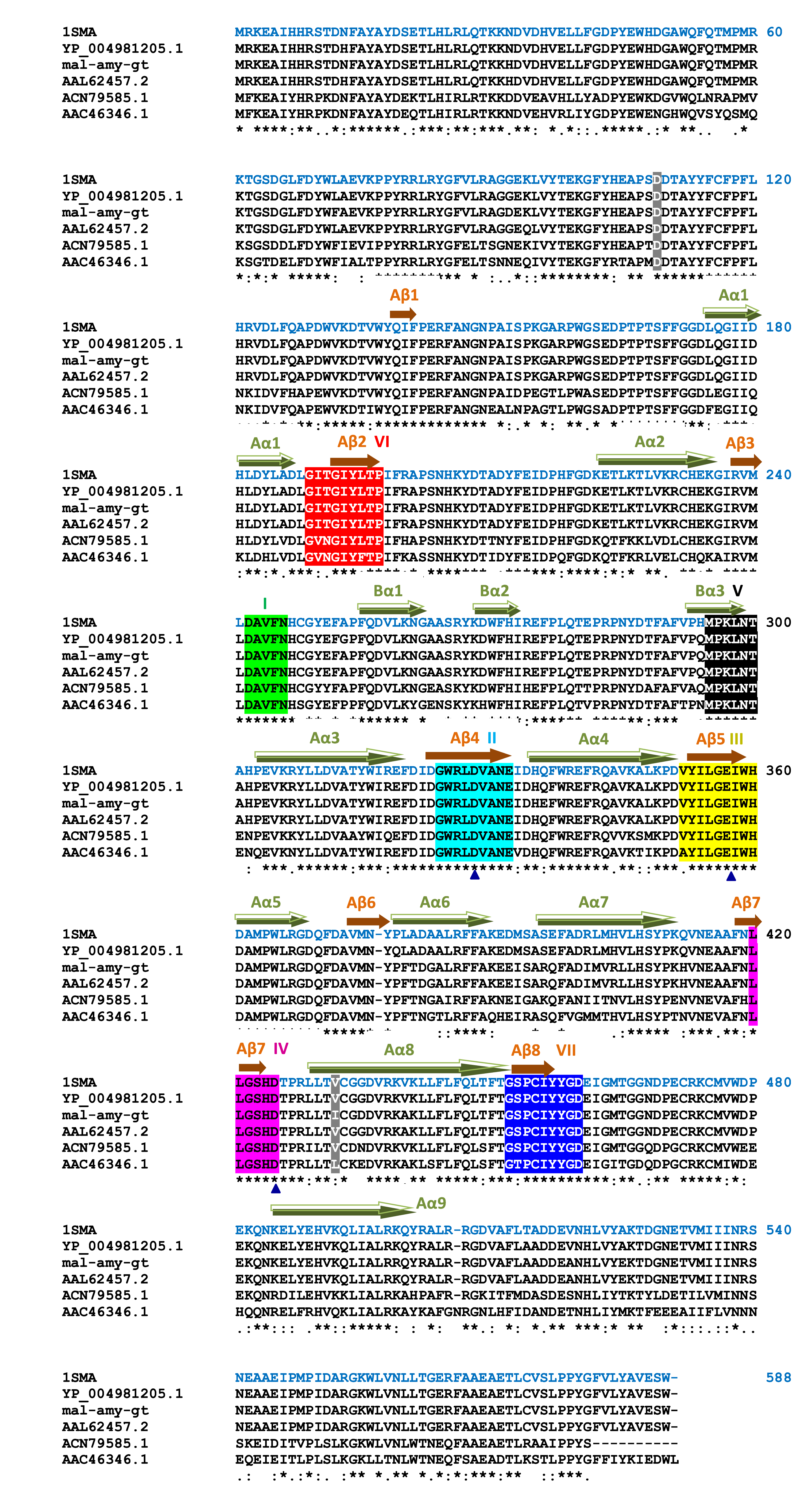

Supplement: Figure S2 — Multiple sequence alignment of in silico translated Gt-Mamy with the other closely related enzymes [G. thermoleovorans CCB_US3_UF5 maltogenic amylase (GenBank ID YP_004981205.1), Thermus sp. YBJ-1 α-cyclodextrinase (GenBank ID AAL62457.2), G. caldoxylosilyticus maltogenic amylase (GenBank ID ACN79585.1), G. stearothermophilus maltogenic amylase (BSMA, GenBank ID AAC46346.1)]. Highlightened regions indicate the seven conserved regions of the α-amylase family. Catalytically active residues are marked by a blue triangle. α-helices and β-strands are indicated by arrows (α-helices by green arrow and β-strands by orange arrows). PDB ID of the template is 1SMA (Thermus sp. IM6501 maltogenic amylase). [Note: 1. Abbreviations: α (α-helix), β (β-strand); 2. ‘*’ indicates the residues in the column that are identical in all sequences, ‘:’ indicates conserved substitutions, ‘.’ indicates semi-conserved substitutions]. GenBank IDs for gt-Mamy and Gt-Mamy are JQ999960.1 and AFM43699.1, respectively. (TIF) [file pone.0073612.s002.tif]

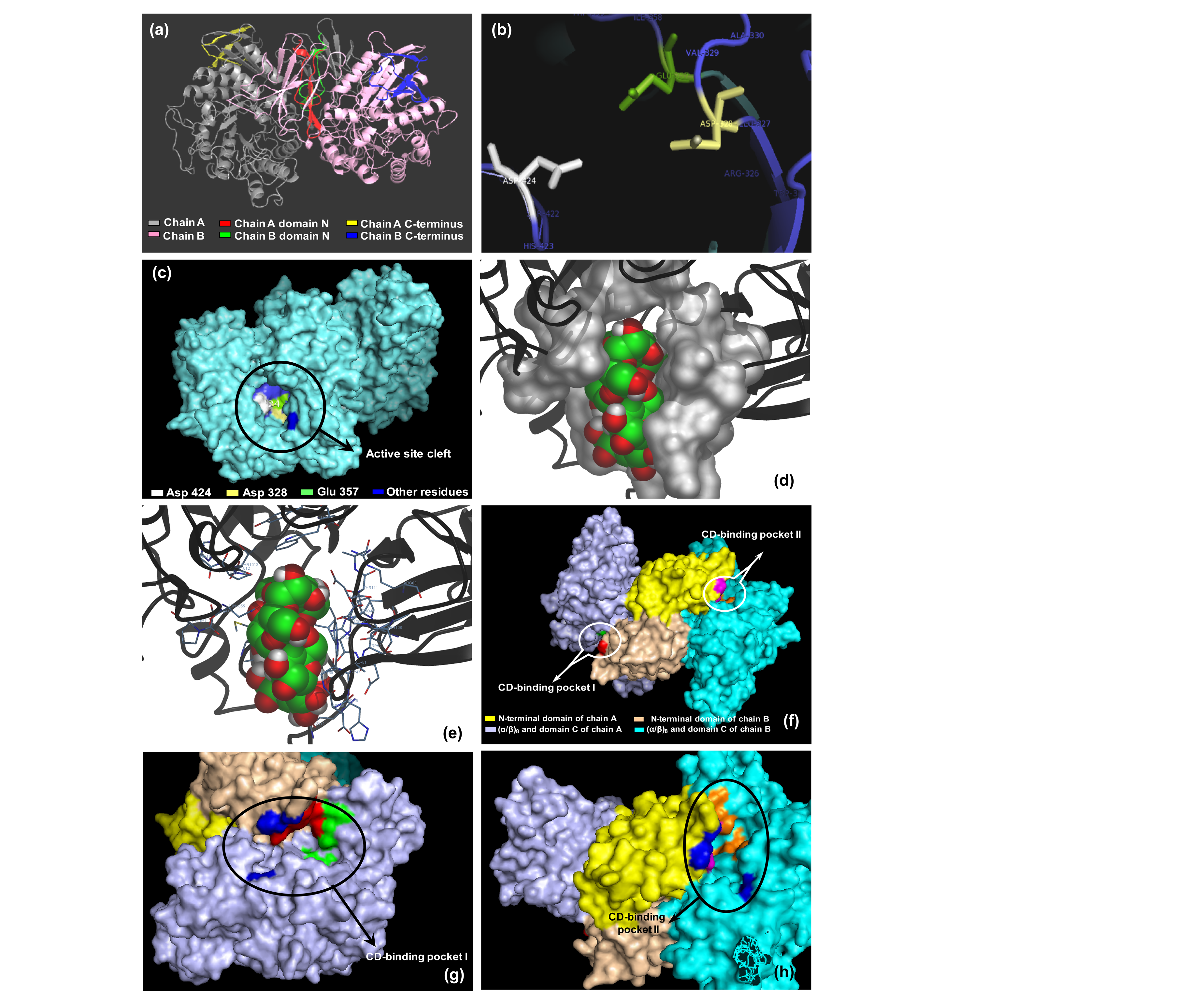

Supplement: Figure S3 — Homology modelling and molecular docking. (a) Quaternary structure of Gt-Mamy showing two chains, A and B; (b) Catalytic residues of Gt-Mamy; (c) Active-site cleft of Gt-Mamy; (d) Molecular docking of β-cyclodextrin to Gt-Mamy (residues that show direct interaction with β-cyclodextrin are shown as surface); (e) Docked sites of β-cyclodextrin binding; (f) Dimeric Gt-Mamy with two β-cyclodextrin binding pockets; (g) Close view of β-cyclodextrin binding pocket I; (h) Close view of β-cyclodextrin binding pocket II. (TIF) [file pone.0073612.s003.tif]
